# Comparison of Outcomes After Primary Laparoscopic Versus Open Approach for T1b/T2 Gallbladder Cancer

**DOI:** 10.3389/fonc.2021.758319

**Published:** 2021-10-28

**Authors:** Jiasheng Cao, Yong Wang, Bin Zhang, Jiahao Hu, Win Topatana, Shijie Li, Sarun Juengpanich, Ziyi Lu, Xiujun Cai, Mingyu Chen

**Affiliations:** ^1^ Department of General Surgery, Sir Run-Run Shaw Hospital, Zhejiang University, Hangzhou, China; ^2^ Zhejiang University School of Medicine, Zhejiang University, Hangzhou, China

**Keywords:** gallbladder cancer (GBC), primary laparoscopic approach, open approach, perioperative outcomes, long-term outcomes

## Abstract

**Objectives:**

The primary laparoscopic approach (PLA) for T1b/T2 gallbladder cancer (GBC) remains contradicted. We aimed to compare the perioperative and long-term outcomes after PLA versus open approach (OA) for T1b/T2 GBC.

**Methods:**

Patients with resected T1b/T2 GBC were selected from our hospital between January 2011 and August 2018. Overall survival (OS), disease-free survival (DFS), and several secondary outcomes were used to evaluate safety and effectiveness. Subgroup analyses were performed to identify significant risk factors for OS/DFS in GBC patients undergoing PLA/OA.

**Results:**

A total of 114 patients who underwent OA (n = 61) or PLA (n = 53) were included in the study. The percent of PLA cases was increased over time from 40.0% in 2011 to 70.0% in 2018 (*p* < 0.05). There was no significant difference in OS [hazard ratio (HR), 1.572; 95% confidence interval (CI), 0.866–2.855; *p* = 0.13] and DFS (HR, 1.225; 95% CI, 0.677–2.218; *p* = 0.49). No significance was found for intraoperative drainage placement (*p* = 0.253), intraoperative blood loss (*p* = 0.497), operation time (*p* = 0.105), postoperative hospitalization (*p* = 0.797), positive LNs (*p* = 0.494), total harvested LNs (*p* = 0.067), and recurrence rates (*P* = 0.334). Subgroup analyses demonstrated no significance of conversion rates after PLA (all *p* > 0.05). Patients undergoing PLA with good/poor OS would have similar recurrence rates (*p* = 0.402). Positive LNs (*p* = 0.032) and tumor differentiation (*p* = 0.048) were identified as risk factors for OS after PLA, while positive LNs (*p* = 0.005) was identified for OS after OA. Moreover, age (*p* = 0.013), gallbladder stone (*p* = 0.008), tumor size (*p* = 0.028), and positive LNs (*p* = 0.044) were potential risk factors for DFS after OA.

**Conclusions:**

PLA for T1b/T2 GBC was comparable to OA in terms of perioperative and long-term outcomes. Less positive LNs and well-differentiated tumors were independent predictors for better OS after PLA, and less positive LNs were also identified for better OS after OA. Additionally, younger age, without gallbladder stone, smaller tumor size, and less positive LNs were potential risk factors for better DFS after OA.

## Introduction

Gallbladder cancer (GBC), which is the most common type of biliary tract malignancy, has a high mortality and a poor dismal prognosis (1–4). Due to the lack of optimal treatment, GBC is considered as a highly lethal disease on the basis of depth and stage of tumor invasion with a 5-year survival of advanced tumors less than 5% (5). According to the 8th American Joint Committee on Cancer (AJCC) Staging Manual (6), simple cholecystectomy is selected for patients with Tis or T1a, while extended/radical cholecystectomy, including removal of adjacent liver parenchyma, resection of the common bile duct, and portal lymphadenectomy, is performed on patients with a histological stage greater than T1b (7).

With the development of advanced surgical devices and accumulation of clinical experience, the application of the laparoscopic approach (LA) has proved its oncologic feasibility and safety in general surgery fields, including liver cancer, gastric cancer, and colon cancer (8–10). Currently, LA has also been utilized for the treatment of GBC. Previous studies have reported favorable long-term outcomes of LA for early GBC (11, 12). For more advanced GBC such as T1b/T2, although several studies showed that the application of LA did not influence the prognosis adversely on the basis of a complete oncologic resection (13–15), there is controversy on whether to choose the primary laparoscopic approach (PLA) or open approach (OA) for T1b/T2 GBC patients. Moreover, they failed to identify risk factors in patients undergoing both two approaches.

The objective of the study was to compare the perioperative and long-term outcomes after PLA versus OA for T1b/T2 GBC patients. Furthermore, we also aimed to identify significant risk factors in patients undergoing different types of resection.

## Materials and Methods

### Study Population

Medical databases of consecutive patients with GBC from January 2011 to August 2018 were retrospectively collected. Patients were selected and included in the study according to the inclusion criteria: (1) age between 18 and 80 years; (2) preoperative imaging diagnosis of GBC and postoperative histopathologic confirmation of T1b/T2 GBC according to the 8th AJCC Staging Manual (6); (3) patients who underwent PLA or OA with radical resection; (4) without other malignancies; and (5) postoperative follow-up was available (≥3 months). Exclusion criteria included (1) insufficient baseline data; (2) without liver resection or lymph nodes (LNs) dissection; (3) positive resection margin; and (4) palliative surgery.

### Baseline Characteristics and Primary/Secondary Outcomes

Patient data on baseline characteristics were collected, including demographic data [age, gender, body mass index (BMI), smoking, and diabetes mellitus (DM)], biliary tract disease-related data (preoperative jaundice and gallbladder stone), tumor features [preoperative carbohydrate antigen 19-9 (CA19-9), preoperative carcinoembryonic antigen (CEA), tumor size, T stage, positive LNs, total harvested LNs, and tumor differentiation], and postoperative adjuvant treatment. Adjuvant therapy included supportive care, chemotherapy, radiotherapy, chemoradiotherapy, targeted therapy, immunotherapy, and traditional medicine therapy within 3 months postoperatively.

Overall survival (OS) and disease-free survival (DFS) were the primary outcomes of the study. We defined OS as the time from operation for GBC until death or the recent follow-up. Furthermore, DFS was calculated as the time interval between resection for GBC and tumor recurrence/relapse or the recent follow-up. Based on the latest outpatient medical records or regular telephone follow-up (every 3 months in postoperative follow-up regularly), the related follow-up data would be obtained. The secondary outcomes included intraoperative drainage placement, intraoperative blood loss, operation time, and postoperative hospitalization, positive LNs, total harvested LNs, conversion rates, and recurrence rates.

### Subgroup Analysis

Considering the median OS of the OA group as a cutoff, the PLA group was divided into “good OS” group (≥ median OS of OA) and “poor OS” group (< median OS of OA). Subgroup analyses using univariable (*p* < 0.1) and consequent multivariable (*p* < 0.05) logistic regression were performed to identify significant risk factors for OS in GBC patients undergoing PLA. Similarly, the PLA group was also divided into “good DFS” group (≥ median DFS of OA) and “poor DFS” group (< median DFS of OA). Subgroup analysis was also performed to identify potential risk factors for DFS in GBC patients undergoing PLA, using univariable (*p* < 0.1) and multivariable logistic regression (*p* < 0.05). We compared conversion rates after PLA between “good OS” group and “poor OS” group, and “good DFS” group and “poor DFS” group, respectively. Moreover, the comparison of recurrence rates after PLA between “good OS” group and “poor OS” group was performed.

Meanwhile, the OA group was divided into “good OS” group (≥ median OS of PLA) and “poor OS” group (< median OS of PLA) based on the cutoff of the median OS of the PLA group. Subgroup analyses using univariable (*p* < 0.1) and consequent multivariable (*p* < 0.05) logistic regression were performed to identify significant risk factors for OS in GBC patients undergoing OA. Similarly, the OA group was also divided into “good DFS” group (≥ median DFS of PLA) and “poor DFS” group (< median DFS of PLA). Furthermore, subgroup analysis was also performed to identify potential risk factors for DFS in GBC patients undergoing OA, using univariable (*p* < 0.1) and multivariable logistic regression (*p* < 0.05). We further compared recurrence rates after OA between “good OS” and “poor OS” group.

### Statistical Analysis

Categorical variables were reported as frequency and percentage, and continuous variables were reported as median and range or means and standard deviations. Categorical variables were assessed between two groups by the χ^2^ test, and continuous variables were compared and analyzed by the Wilcoxon rank-sum test or Student’s *t*-test. Kaplan–Meier survival analysis was conducted to analyze the difference in OS and DFS between two groups. All analyses were performed by SPSS version 20.0 (IBM SPSS, Inc., Chicago, IL) and R version 4.0.4 (R Foundation for Statistical Computing, Vienna, Austria) with the “survival” package. *p* < 0.05 was considered statistically significant.

## Results

**Figure 1:**
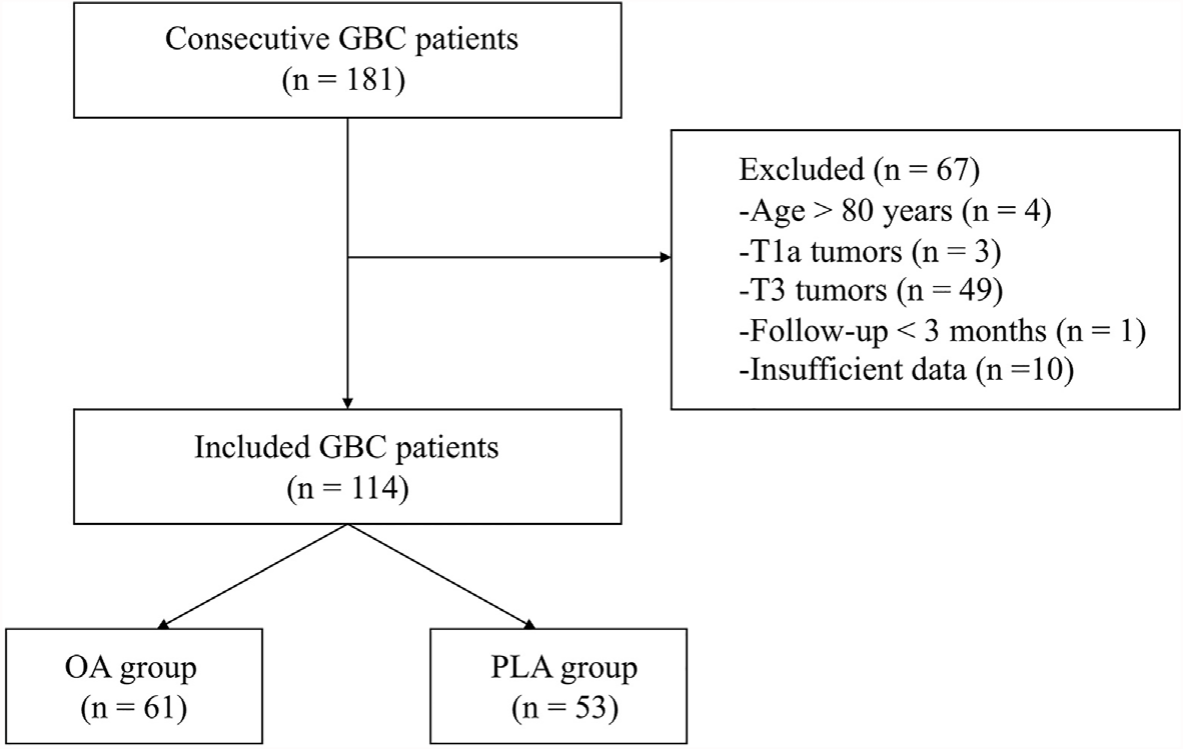
A flow diagram of the included patients. GBC, gallbladder cancer; OA, open approach; PLA, primary laparoscopic approach.

Medical databases of 181 consecutive GBC patients were obtained. After excluding patients aged >80 (n = 4), with non-T1b/T2 tumors (n = 52, [3 T1a + 49 T3]), with follow-up less than 3 months (n = 1), and with insufficient data (n = 10), a total of 114 GBC patients, consisting of 61 patients in the OA group (n =61) and 53 patients in the PLA group (n = 53), were included in the study (**Figure 1**).

### PLA Cases Over Time

**Figure 2:**
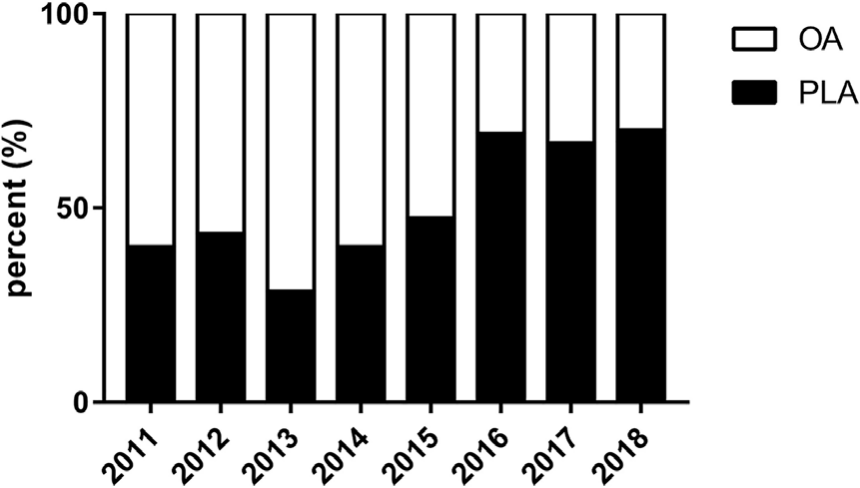
Percent of PLA cases over time for GBC. GBC, gallbladder cancer; OA, open approach; PLA, primary laparoscopic approach.

The percent of PLA cases for T1b/T2GBC was increased over time from 40.0% in 2011 to 70.0% in 2018 (*p* < 0.05) (**Figure 2**). Specifically, the PLA percent was started with 40.0% in 2011, and then it was descended to the lowest level of 28.6% in 2013. Between the years of 2013 and 2015, the PLA percent was increased steadily at around 10% annually. Moreover, since 2016, the PLA percent was maintained at an unprecedented high level of approximately 70%.

### Baseline Characteristics

During the study period, 61 GBC patients received radical resection by OA while PLA of radical resection were performed on 53 GBC patients. The baseline characteristics (demographic data, biliary tract disease-related data, tumor features, and postoperative adjuvant treatment) of the 114 included GBC patients are summarized in **Table 1**. An adequate balance was observed between the OA group and PLA group for all variables (all *p* > 0.05).

**Table 1:** Baseline characteristics of the included GBC patients.

Variables	All patients (n = 114)	OA group (n = 61)	PLA group (n = 53)	*p* value
**Demographic data**				
Age (years)	62 (39–79)	64 (39–79)	61 (48–77)	0.674
Gender ratio (male: female)	32: 82	14: 47	18: 35	0.192
BMI ≥ 25 kg/m^2^	41 (36.0)	27 (44.3)	14 (26.4)	0.053
Smoking	7 (6.1)	5 (8.2)	2 (3.8)	0.327
DM	6 (5.3)	3 (4.9)	3 (5.7)	0.859
**Biliary tract disease-related data**				
Preoperative jaundice	0	0	0	–
Gallbladder stone	47 (41.2)	29 (47.5)	18 (34.0)	0.142
**Tumor features**				
Preoperative CA19-9 (≤37 U/ml)	85 (74.6)	46 (75.4)	39 (73.6)	0.823
Preoperative CEA (≤5 ng/ml)	99 (86.8)	55 (90.2)	44 (83.0)	0.260
Tumor size (cm)				0.123
≤1	22 (19.3)	16 (26.2)	6 (11.3)	
1–3	57 (50.0)	27 (44.3)	30 (56.6)	
>3	35 (30.7)	18 (29.5)	17 (32.1)	
T stage				0.597
T1b	8 (7.0)	5 (8.2)	3 (5.7)	
T2	106 (93.0)	56 (91.8)	50 (94.3)	
Positive LNs	0 (0–6)	0 (0–5)	0 (0–6)	0.494
Total harvested LNs	7 (1–42)	8 (1–42)	6 (1–16)	0.067
Tumor differentiation				0.505
Well	54 (47.4)	32 (52.5)	22 (41.5)	
Moderately	23 (20.2)	11 (18.0)	12 (22.6)	
Poorly	37 (32.5)	18 (29.5)	19 (35.8)	
**Postoperative adjuvant treatment**				0.131
Supportive care	76 (66.7)	46 (75.4)	30 (56.6)	
Chemotherapy	11 (9.6)	3 (4.9)	8 (15.1)	
Radiotherapy	2 (1.8)	0 (0)	2 (3.8)	
Chemoradiotherapy	21 (18.4)	10 (16.4)	11 (20.8)	
Targeted therapy	1 (0.9)	0 (0)	1 (1.9)	
Immunotherapy	0 (0)	0 (0)	0 (0)	
Traditional medicine therapy	3 (2.6)	2 (3.3)	1 (1.9)	

GBC, gallbladder cancer; OA, open approach; PLA, primary laparoscopic approach; BMI, body mass index; DM, diabetes mellitus; CA19-9, carbohydrate antigen 19-9; CEA, carcinoembryonic antigen; LNs, lymph nodes.

### Primary and Secondary Outcomes

**Figure 3:**
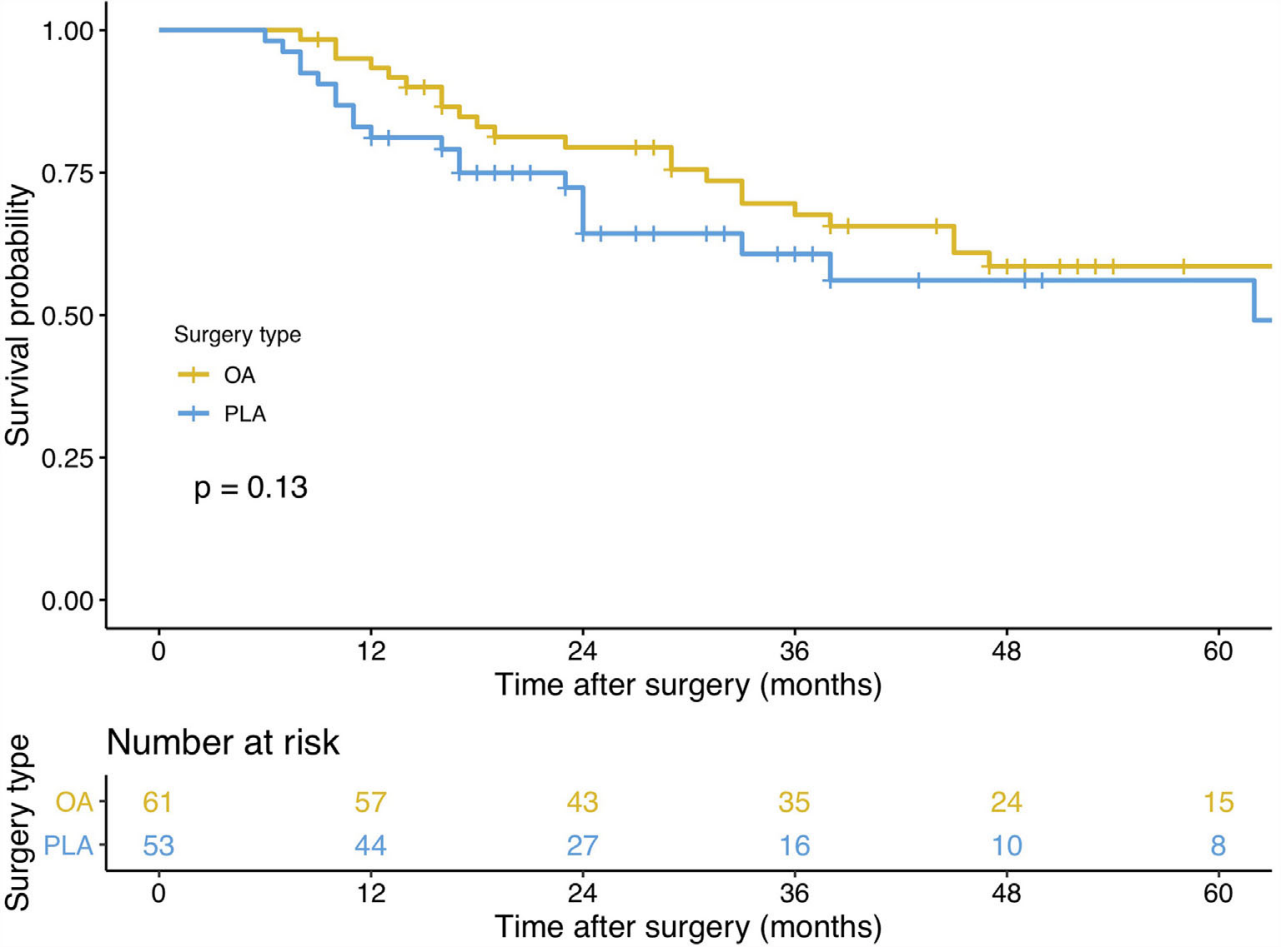
A comparison of overall survival of primary outcomes after OA or PLA in GBC patients. OA, open approach; PLA, primary laparoscopic approach; GBC, gallbladder cancer.

**Figure 4:**
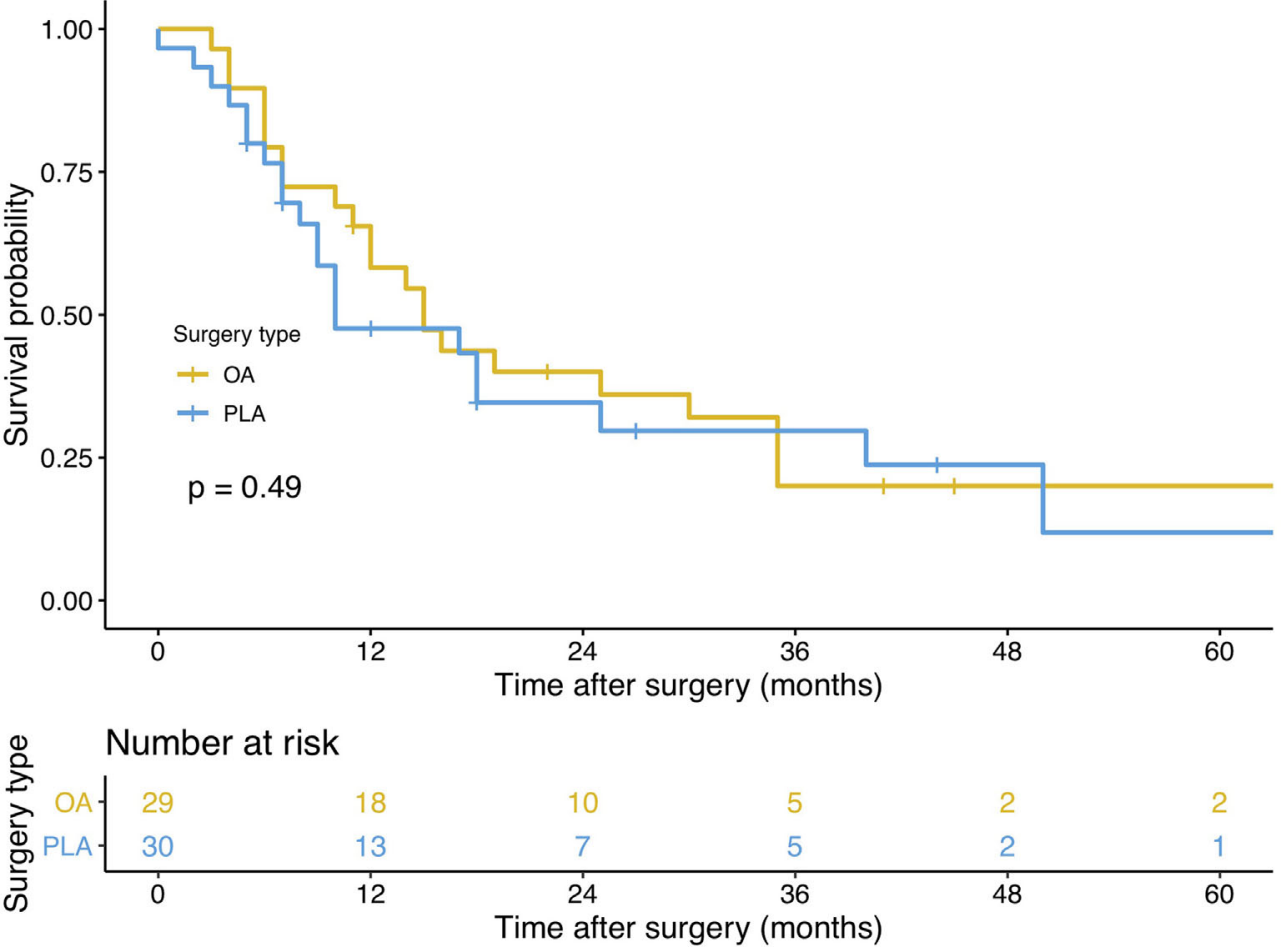
A comparison of disease-free survival of primary outcomes after OA or PLA in GBC patients. OA, open approach; PLA, primary laparoscopic approach; GBC, gallbladder cancer.

**Figure 5:**
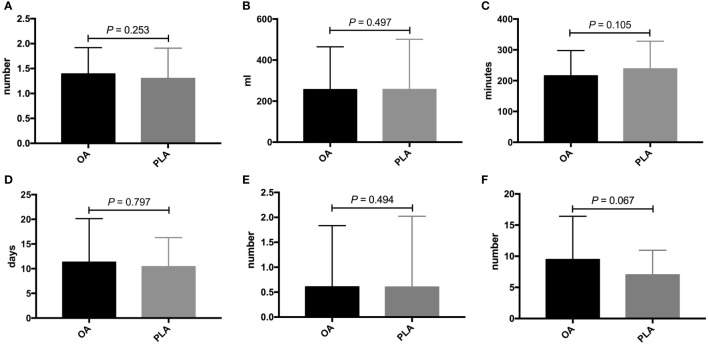
Comparisons of secondary outcomes after OA or PLA in GBC patients. The difference of **(A)** intraoperative drainage placement, **(B)** intraoperative blood loss, **(C)** operation time, **(D)** postoperative hospitalization, **(E)** positive LNs, and **(F)** total harvested LNs. OA, open approach; PLA, primary laparoscopic approach; GBC, gallbladder cancer; LNs, lymph nodes.

PLA compared with OA demonstrated no significant benefit on OS (hazard ratio [HR], 1.572; 95% confidence interval [CI], 0.866–2.855; *p* = 0.13, **Figure 3**) and DFS (HR, 1.225; 95% CI, 0.677–2.218; *p* = 0.49, **Figure 4**). In addition, the number of intraoperative drainage placement was less after PLA, but no significant difference was observed between both groups (PLA 1.3 *vs*. OA 1.4, *p* = 0.253, **Figure 5A**). PLA would not cause significantly more intraoperative blood loss (PLA 257.0 ml *vs*. OA 256.2 ml, *p* = 0.497, **Figure 5B**) and would not take longer operations time (PLA 238.4 min *vs*. OA 215.7 min, *p* = 0.105, **Figure 5C**) in GBC patients. Patients undergoing PLA had less postoperative hospitalization than OA, although there was no significant difference between PLA and OA groups (PLA 10.4 days *vs*. OA 11.3 days, *p* = 0.797, **Figure 5D**). As for LN yield, no significant difference was demonstrated in the number of positive LNs (PLA 0 *vs*. OA 0, *p* = 0.494, **Figure 5E**) and total harvested LNs (PLA 7 *vs*. OA 8, *p* = 0.067, **Figure 5F**). Meanwhile, no significance was shown in recurrence rates between the PLA group and OA group (PLA 56.6% *vs*. OA 47.5%, *p* = 0.334, **Figure S1A**).

### Subgroup Analysis

The exploratory subgroup analysis was performed to identify potential risk factors for OS in GBC patients undergoing PLA (n = 53) (**Table 2**). On the basis of univariable analysis, three variables with a *p* value less than 0.1, including smoking (*p* = 0.045), positive LNs (*p* < 0.001), and tumor differentiation (*p* = 0.006) were selected and taken into multivariate analysis with the Cox proportional hazards regression model. After multivariate analysis, two variables including positive LNs (*p* = 0.032) and tumor differentiation (*p* = 0.048) were identified as the independent risk factors for OS after PLA. The potential risk factors for DFS in GBC patients undergoing PLA (n = 30) were also identified (**Table 3**). After univariable analysis, two variables including positive LNs (*p* = 0.065) and tumor differentiation (*p* = 0.069) were taken into multivariate analysis. However, both two variables had no significant difference on DFS of GBC patients after PLA. Moreover, as for the conversion rates after PLA, there was no significant difference between “good OS” group and “poor OS” group (good OS 18.2% *vs*. poor OS 26.2%, *p* = 0.583, **Figure S2A**), and between “good DFS” group and “poor DFS” group (good DFS 18.2% *vs*. poor DFS 26.3%, *p* = 0.612, **Figure S2B**). Meanwhile, patients undergoing PLA with good OS would not have significantly lower recurrence rates than those with poor OS (good OS 45.5% *vs*. poor OS 59.5%, *p* = 0.402, **Figure S1B**).

**Table 2:** Potential risk factors for OS in GBC patients undergoing PLA based on univariable and multivariable analyses.

Variables	Poor OS (n = 42)	Good OS (n = 11)	Univariable analysis		Multivariable analysis	
			HR [95%CI]	*p* value	HR [95%CI]	*p* value
**Demographic data**						
Age (years)	61.5 (48–77)	53 (48–72)	1.001 [0.956–1.048]	0.966		
Gender ratio (male: female)	15:27	3: 8	1.262 [0.517–3.076]	0.609		
BMI ≥ 25 kg/m^2^	11 (26.2)	3 (27.3)	1.104 [0.429–2.842]	0.838		
Smoking	2 (4.8)	0	4.673 [1.032–21.159]	0.045*	1.706 [0.309–9.408]	0.540
DM	3 (7.1)	0	1.186 [0.157–8.962]	0.869		
**Biliary tract disease-related data**						
Preoperative jaundice	0	0	–	–		
Gallbladder stone	16 (38.1)	2 (18.2)	0.687 [0.252–1.869]	0.462		
**Tumor features**						
Preoperative CA19-9 (≤37 U/ml)	31 (73.8)	8 (72.7)	0.566 [0.236–1.356]	0.202		
Preoperative CEA (≤5 ng/ml)	36 (85.7)	8 (72.7)	0.619 [0.241–1.588]	0.318		
Tumor size (cm)			0.998 [0.508–1.961]	0.995		
≤1	5 (11.9)	1 (9.1)				
1–3	25 (59.5)	5 (45.5)				
>3	12 (28.6)	5 (45.5)				
T stage			–	–		
T1b	3 (7.1)	0				
T2	39 (92.9)	11 (100)				
Positive LNs	0.691 ± 1.554	0.273 ± 0.647	1.531 [1.215–1.929]	<0.001*	1.349 [1.027–1.772]	0.032*
Total harvested LNs	7.476 ± 3.776	5.273 ± 4.245	1.026 [0.922–1.141]	0.644		
Tumor differentiation			2.080 [1.233–3.510]	0.006*	1.771 [1.006–3.120]	0.048*
Well	14 (33.3)	8 (72.7)				
Moderately	10 (23.8)	2 (18.2)				
Poorly	18 (42.9)	1 (9.1)				
**Postoperative adjuvant treatment**			0.989 [0.741–1.320]	0.938		
Supportive care	23 (54.8)	7 (63.6)				
Chemotherapy	8 (19.0)	0				
Radiotherapy	0	2 (18.2)				
Chemoradiotherapy	9 (21.4)	2 (18.2)				
Targeted therapy	0	0				
Immunotherapy	1 (2.4)	0				
Traditional medicine therapy	1 (2.4)	0				

OS, overall survival; GBC, gallbladder cancer; PLA, primary laparoscopic approach; HR, hazards ratio; CI, confidence interval; BMI, body mass index; DM, diabetes mellitus; CA19-9, carbohydrate antigen 19-9; CEA, carcinoembryonic antigen; LNs, lymph nodes.

*The bold values meant P < 0.05, indicating significant difference.

**Table 3:** Potential risk factors for DFS in GBC patients undergoing PLA based on univariable and multivariable analyses.

Variables	Poor DFS (n = 19)	Good DFS (n = 11)	Univariable analysis		Multivariable analysis	
			HR [95%CI]	*p* value	HR [95%CI]	*p* value
**Demographic data**						
Age (years)	61 (48–77)	62 (48–76)	1.008 [0.962–1.057]	0.728		
Gender ratio (male: female)	6: 13	6: 5	0.744 [0.301–1.836]	0.520		
BMI ≥ 25 kg/m^2^	6 (31.6)	2 (18.2)	1.215 [0.463–3.191]	0.692		
Smoking	1 (5.3)	1 (9.1)	1.855 [0.420–8.188]	0.414		
DM	2 (10.5)	0	1.040 [0.136–7.927]	0.970		
**Biliary tract disease-related data**						
Preoperative jaundice	0	0	–	–		
Gallbladder stone	6 (31.6)	3 (27.3)	0.900 [0.326–2.486]	0.839		
**Tumor features**						
Preoperative CA19-9 (≤37 U/ml)	13 (68.4)	6 (54.5)	0.943 [0.384–2.317]	0.899		
Preoperative CEA (≤5 ng/ml)	15 (78.9)	8 (72.7)	0.875 [0.335–2.288]	0.785		
Tumor size (cm)			0.938 [0.476–1.852]	0.855		
≤1	1 (5.3)	1 (9.1)				
1–3	12 (63.2)	5 (45.5)				
>3	6 (31.6)	5 (45.5)				
T stage			–	–		
T1b	0	0				
T2	19 (100.0)	11 (100.0)				
Positive LNs	0 (0–6)	0 (0–2)	1.275 [0.985–1.649]	0.065*	1.178 [0.892–1.557]	0.248
Total harvested LNs	6 (2–16)	4 (1–13)	1.079 [0.974–1.196]	0.143		
Tumor differentiation			1.749 [0.958–3.193]	0.069*	1.564 [0.826–2.962]	0.170
Well	3 (15.8)	5 (45.5)				
Moderately	3 (15.8)	4 (36.4)				
Poorly	13 (68.4)	2 (18.2)				
**Postoperative adjuvant treatment**			0.900 [0.644–1.259]	0.540		
Supportive care	9 (47.4)	7 (63.6)				
Chemotherapy	6 (31.6)	0				
Radiotherapy	0	1 (9.1)				
Chemoradiotherapy	4 (21.1)	3 (27.3)				
Targeted therapy	0	0				
Immunotherapy	0	0				
Traditional medicine therapy	0	0				

DFS, disease-free survival; GBC, gallbladder cancer; PLA, primary laparoscopic approach; HR, hazards ratio; CI, confidence interval; BMI, body mass index; DM, diabetes mellitus; CA19-9, carbohydrate antigen 19-9; CEA, carcinoembryonic antigen; LNs, lymph nodes.

*The bold values meant P < 0.1, indicating significant difference.

In the OA group (n = 61), another subgroup analysis was also conducted to identify potential risk factors for OS in GBC patients (**Table S1**). After univariable analysis, two variables with a *p* value less than 0.1, including preoperative CEA (*p* = 0.066) and positive LNs (*p* = 0.039) were selected for the consequent multivariate analysis. Moreover, positive LNs (*p* = 0.005) were identified as the independent risk factor for OS after OA. Additionally, we identified potential risk factors for DFS in patients undergoing OA (**Table S2**). Based on the univariable analysis, four variables consisting age (*p* = 0.005), gallbladder stone (*p* = 0.046), tumor size (*p* = 0.015), and positive LNs (*p* = 0.057) were entered into multivariate analysis. Consequently, age (*p* = 0.013), gallbladder stone (*p* = 0.008), tumor size (*p* = 0.028), and positive LNs (*p* = 0.044) were identified as potential risk factors for DFS in GBC patients. Notably, patients after OA in the “good OS” group would have significantly lower recurrence rates than those in the “poor OS” group (good OS 37.2% *vs*. poor OS 72.2%, *p* = 0.013, **Figure S1C**).

## Discussion

In this study, PLA was not inferior to OA regarding OS, DFS, intraoperative drainage placement, intraoperative blood loss, operation time, postoperative hospitalization, number of positive LNs, number of total harvested LNs, and recurrence rates. Moreover, subgroup analyses identified that less positive LNs and well-differentiated tumors were independent predictors for better OS after PLA, and less positive LNs were also identified for better OS after OA. Additionally, younger age, without gallbladder stone, smaller tumor size, and less positive LNs were potential risk factors for better DFS after OA.

PLA was not recommended for T1b/T2 GBC patients based on the previous Japanese Association of Biliary Surgery Guidelines (16). Notably, tumor exposure and implantation may happen during the intraoperative procedure, which was caused by the high risk of gallbladder perforation and bile spillage. Moreover, port-site recurrences after PLA were reported in GBC patients due to the technical shortcomings including nonuse of retrieval bags and poor surgeon-related operation skills (17). Meanwhile, PLA was not primarily chosen for GBC regarding the safety and feasibility of the approach. However, with the surgical techniques developed, similar oncological outcomes may be achieved in both of the PLA and OA for gastric carcinoma (18), colorectal carcinoma (19), and GBC patients (14).

Significant progress was achieved in laparoscopic resection for GBC in the year of 2011, which was approximately the turning point of the new approach (20). Laparoscopic resection for GBC is technically challenging, which requires advanced laparoscopic skills, especially when performing segment IVb/V resection or wedge resection with a complete lymphadenectomy for T2 GBC (21). There are concerns that LA may not meet the standards of OA, leading to inadequate resection, tumor cell dissemination, and poor prognosis of GBC (22). However, laparoscopic liver resection, including major and minor hepatectomy, has been confirmed feasible (8). Moreover, Agarwal et al. (13) concluded that an R0 resection with lymphadenectomy could be accomplished in T1b-T3 GBC patients without gallbladder perforation and bile spillage. Notably, developments in laparoscopic surgical instrumentation and technical innovation have contributed to the appropriate quality of extended resection for T1b/T2 GBC (23–25). Hepatectomy was performed to facilitate R0 resection by using preoperative three-dimensional reconstruction (26), intraoperative ultrasonography guidance (27), and intraoperative laparoscopic Glissonian approach (28). Although there is no consensus about lymphadenectomy extension for T1b/T2 GBC, hepatoduodenal ligament LN resection with and without extraregional LN dissection are recommended for T1b/T2 GBC patients, respectively (29, 30).

One of the strongest predictors among GBC patients is the regional LN status (31), and patients have worse prognosis with an increasing number of positive LNs. The study identified that the potential risk factor for OS in T1b/T2 GBC patients undergoing PLA was the number of positive resected LNs. The 8th AJCC staging recommended that at least six LNs should be harvested and evaluated (32, 33), and PLA is similar to OA with respect to the resection of total LNs in the present study. After achieving a systematic and complete resection, the accurate prediction of the prognosis of T1b/T2 GBC patients is associated with the LN staging, which is based on the number of positive ones (34, 35). Tumor immune responses would be the mechanism for the number of positive LNs affecting prognosis of GBC after surgery. Similar to colorectal cancer (36), the benefits associated with less positive LNs may reflect weaker effects of LN micrometastasis and higher host lymphocytic response to the GBC, which meant that more infiltrating dendritic cells correlated with fewer further metastasis to LNs. Moreover, dendritic cells were found to significantly correlate with OS (37). Besides the assessment of LN status after dissection, further improvement in identifying positive LNs from preoperative imaging and increasing the number of positive/total resected LNs is required for better OS for T1b/T2 GBC patients.

Tumor differentiation is another potential risk factor for long-term outcomes in T1b/T2 GBC patients undergoing PLA. Histological tumor differentiation represents the biological characteristics of GBC which tend to positively correlate with tumor aggressiveness. Compared with poorly and moderately differentiated GBC, well-differentiated GBC usually have a glandular structure with less cellular density (38), in which patient pericholecystic infiltration and regional LN enlargement are infrequently observed, leading to poor prognosis. Several studies used tumor differentiation to predict long-term outcomes in GBC patients (39, 40). For example, a nomogram was developed and validated based on clinicopathological factors, such as tumor differentiation, to predict 1-, 3-, and 5-year OS in resected GBC patients (39). Notably, Min et al. (41) have found that the apparent diffusion coefficient value on diffusion-weighted magnetic resonance imaging was significantly associated with tumor differentiation and long-term outcomes after surgery. Despite that tumor differentiation is based on the histopathological results currently, diffusion-weighted magnetic resonance imaging may be utilized for preoperative prediction for tumor differentiation.

There are an increasing number of studies comparing the outcomes of laparoscopic and open radical surgery for GBC patients. Although a latest Chinese single-center study concluded that LA had comparable intraoperative, perioperative, and survival outcomes with OA for incidental GBC patients of T1b/T2, it merely included 50 patients, which meant the sample size was too small to convince surgeons to decide the optimal approach preoperatively (42). Another retrospective study (43) conducted by Hamad et al. demonstrated that GBC patients undergoing radical resection had similar rates of harvested LNs regardless of the operation approach, but the study also included patients before 2011 and no definite surgical strategy was provided. What is more, a meta-analysis, which included seven comparative studies and eight non-comparative studies, confirmed that LA was safe and feasible with comparable operation-related and survival outcomes for T1b/T2 GBC (44). However, different from previous studies, the current study focused on comparing PLA with OA for GBC patients after the year 2011, which is the year of technological innovation, and identifying which patients may benefit most from operation approaches.

The study has several limitations that need to be considered. First, this is a single and retrospective study, whose sample size is too small to provide a high-level evidence. As the baseline characteristics of included patients are balanced between PLA and OA groups, the drawbacks may be partly avoided. Additionally, the specific hepatectomy strategy for T2 GBC patients was not distinguished for further analysis in the study. Whether to choose wedge resection or the more radical segment IVb/V resection for T2 GBC patients remains controversial, and surgeons should rely on surgical skills and patients’ medical records to choose the optimal approach (21). Besides, the study did not concern intraoperative complications (bile duct injury, air embolus, electrolyte/glucose abnormalities, hemodynamic instability, respiratory compromise, and renal dysfunction) and postoperative complications (infection, bile leakage, bleeding, and liver dysfunction), owing that it was focused on exploring and comparing the prognosis of the primary surgical approach for T1b/T2 GBC patients. Therefore, multicenter retrospective or even prospective studies of large sample size should be performed to compare the outcomes of LA and OA for T1b/T2 GBC patients; meanwhile, subgroup analysis of PLA and pure LA would be considered.

In conclusion, PLA was not inferior to OA regarding perioperative outcomes, OS, and DFS for T1b/T2 GBC patients. Less positive LNs and well-differentiated tumors were two independent predictors for better OS after PLA, and less positive LNs were also identified for better OS after OA. Additionally, younger age, without gallbladder stone, smaller tumor size, and less positive LNs were potential risk factors for better DFS after OA.

## Data Availability Statement

The raw data supporting the conclusions of this article will be made available by the authors, without undue reservation.

## Ethics Statement

The studies involving human participants were reviewed and approved by the institutional ethics review board of Sir Run Run Shaw Hospital, Zhejiang University, Hangzhou, Zhejiang Province, China (20210625-30). Written informed consent for participation was not required for this study in accordance with the national legislation and the institutional requirements.

## Author Contributions

JC, YW, BZ, XC, and MC designed the study and collected the data. JH, WT, SL, and SJ analyzed and interpreted the data. JC, YW, BZ, ZL, and MC wrote the manuscript. MC revised the manuscript. All authors contributed to the article and approved the submitted version.

## Funding

This work was supported by the Zhejiang Medical and Health Science and Technology Project (grant number 2019321842), National Natural Science Foundation of China (grant number 81827804), Zhejiang Clinical Research Center of Minimally Invasive Diagnosis and Treatment of Abdominal Diseases (grant number 2018E50003), and Key Research and Development Project of Zhejiang Province (grant number 2018C03083).

## Conflict of Interest

The authors declare that the research was conducted in the absence of any commercial or financial relationships that could be construed as a potential conflict of interest.

## Publisher’s Note

All claims expressed in this article are solely those of the authors and do not necessarily represent those of their affiliated organizations, or those of the publisher, the editors and the reviewers. Any product that may be evaluated in this article, or claim that may be made by its manufacturer, is not guaranteed or endorsed by the publisher.

## Supplementary Material

Supplementary Figure 1 Comparison of recurrence rates in GBC patients **(A)** undergoing PLA or OA, **(B)** undergoing PLA between “good OS” group and “poor OS” group, and **(C)** undergoing PLA between “good DFS” group and “poor DFS” group. GBC, gallbladder cancer; PLA, primary laparoscopic approach; OA, open approach; OS, overall survival; DFS, disease-free survival.

Supplementary Figure 2 Comparisons of conversion rates in GBC patients undergoing PLA **(A)** between “good OS” group and “poor OS” group, and **(B)** between “good DFS” group and “poor DFS” group. GBC, gallbladder cancer; PLA, primary laparoscopic approach; OS, overall survival; DFS, disease-free survival.

## Abbreviations

GBC, gallbladder cancer; AJCC, American Joint Committee on Cancer; LA, laparoscopic approach; PLA, primary laparoscopic approach; OA, open approach; LNs, lymph nodes; BMI, body mass index; DM, diabetes mellitus; CA19-9, carbohydrate antigen 19-9; CEA, carcinoembryonic antigen; OS, overall survival; DFS, disease-free survival; HR, hazard ratio; CI, confidence interval.
