# Supplementary figures and images for: Comparison of Outcomes After Primary Laparoscopic Versus Open Approach for T1b/T2 Gallbladder Cancer
Source: Front Oncol. 2021 Oct 28;11:758319. doi: 10.3389/fonc.2021.758319 (PMC8580936; doi:10.3389/fonc.2021.758319)

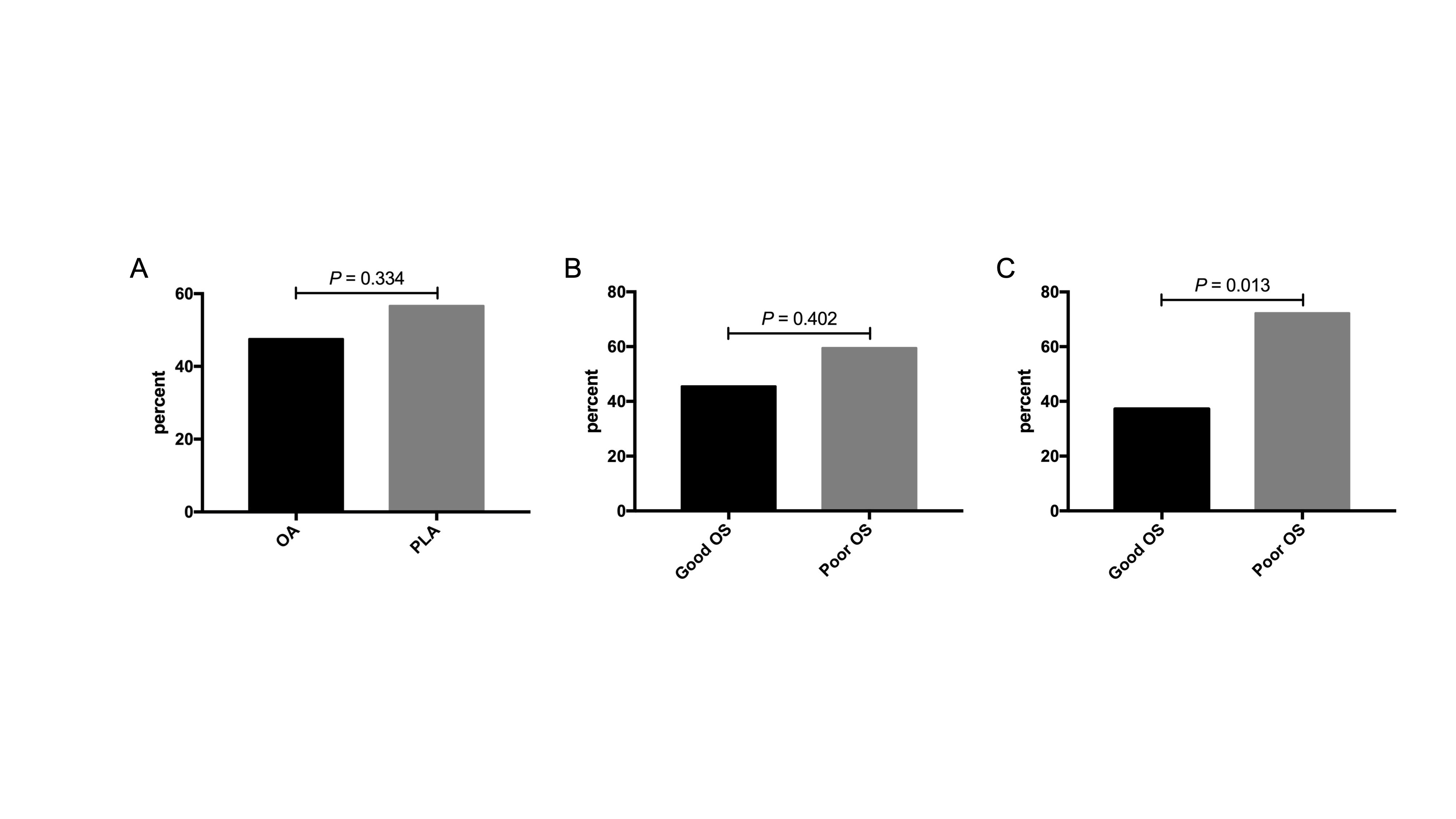

Supplement: Supplementary Figure 1 — Comparison of recurrence rates in GBC patients (A) undergoing PLA or OA, (B) undergoing PLA between “good OS” group and “poor OS” group, and (C) undergoing PLA between “good DFS” group and “poor DFS” group. GBC, gallbladder cancer; PLA, primary laparoscopic approach; OA, open approach; OS, overall survival; DFS, disease-free survival. [file Image_1.jpeg]

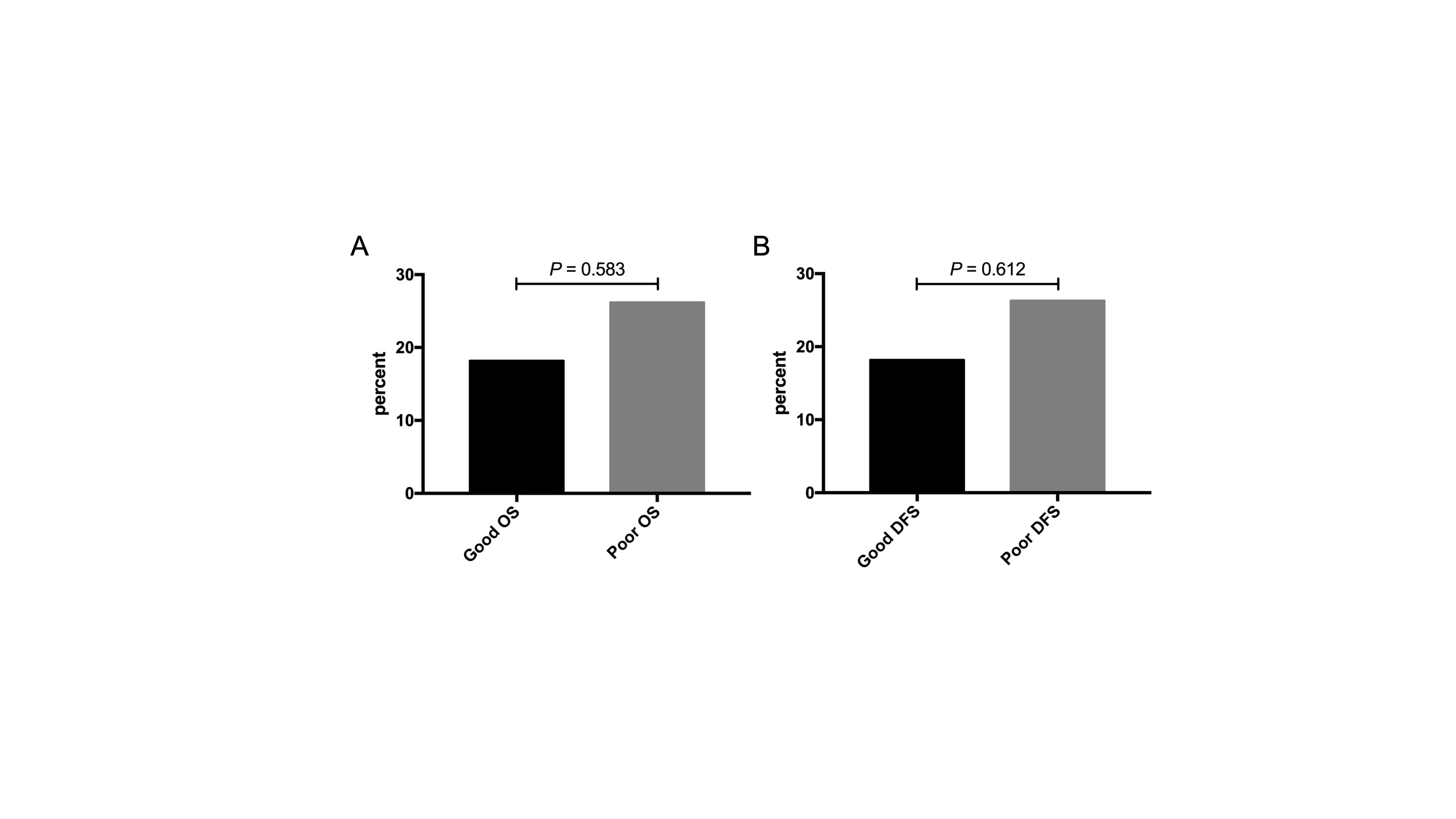

Supplement: Supplementary Figure 2 — Comparisons of conversion rates in GBC patients undergoing PLA (A) between “good OS” group and “poor OS” group, and (B) between “good DFS” group and “poor DFS” group. GBC, gallbladder cancer; PLA, primary laparoscopic approach; OS, overall survival; DFS, disease-free survival. [file Image_2.jpeg]
