# Supplementary material for: Comparison of Outcomes After Primary Laparoscopic Versus Open Approach for T1b/T2 Gallbladder Cancer
Source: Front Oncol. 2021 Oct 28;11:758319. doi: 10.3389/fonc.2021.758319 (PMC8580936; doi:10.3389/fonc.2021.758319)
Supplement: Supplementary file 3 [file Table_1.docx]

| Table S1. Potential risk factors for OS in GBC patients undergoing OA based on univariable and multivariable analysis. | | | | | | |  |
| --- | --- | --- | --- | --- | --- | --- | --- |
| **Variables** | **Poor OS (n = 18)** | **Good OS (n = 43)** | **Univariable analysis** | | **Multivariable analysis** | | |
|  |  |  | **HR [95%CI]** | ***P* value** | **HR [95%CI]** | ***P* value** | |
| **Demographic data** |  |  |  |  |  |  | |
| Age (years) | 69 (42-79) | 60 (39-73) | 1.040  [0.982-1.100] | 0.179 |  |  | |
| Gender ratio (Male : Female) | 6 : 12 | 8 : 35 | 1.477  [0.422-5.168] | 0.542 |  |  | |
| BMI ≥ 25 kg/m^2^ | 11 (61.1) | 21 (48.8) | 1.036  [0.855-1.255] | 0.719 |  |  | |
| Smoking | 0 | 5 (11.6) | 1.722  [0.252-11.782] | 0.579 |  |  | |
| DM | 0 | 3 (7.0) | 0.931  [0.104-8.355] | 0.949 |  |  | |
| **Biliary tract disease-related data** |  |  |  |  |  |  | |
| Preoperative jaundice | 0 | 0 | - | - |  |  | |
| Gallbladder stone | 19 (55.6) | 19 (44.2) | 1.521  [0.514-4.500] | 0.448 |  |  | |
| **Tumor features** |  |  |  |  |  |  | |
| Preoperative CA19-9 (≤ 37 U/ml) | 13 (72.2) | 33 (76.7) | 0.633  [0.170-2.360] | 0.496 |  |  | |
| Preoperative CEA (≤ 5 ng/mL) | 16 (88.9) | 39 (90.7) | 0.310  [0.089-1.079] | **0.066*** | 0.421  [0.142-1.250] | 0.119 | |
| Tumor size (cm) |  |  | 0.773  [0.337-1.771] | 0.543 |  |  | |
| ≤ 1 | 6 (33.3) | 10 (23.3) |  |  |  |  | |
| 1 - 3 | 5 (27.8) | 22 (51.2) |  |  |  |  | |
| > 3 | 7 (38.9) | 11 (25.6) |  |  |  |  | |
| T stage |  |  | - | - |  |  | |
| T1b | 1 (5.6) | 4 (9.3) |  |  |  |  | |
| T2 | 17 (94.4) | 39 (90.7) |  |  |  |  | |
| Positive LNs | 1.000±1.609 | 0.442±1.007 | 1.497  [1.021-2.196] | **0.039*** | 1.425  [1.112-1.826] | **0.005*** | |
| Total harvested LNs | 7.611±4.754 | 10.256±7.582 | 0.980  [0.911-1.056] | 0.602 |  |  | |
| Tumor differentiation |  |  | 1.582  [0.827-3.027] | 0.166 |  |  | |
| Well | 8 (44.4) | 24 (55.8) |  |  |  |  | |
| Moderately | 3 (16.7) | 8 (18.6) |  |  |  |  | |
| Poorly | 7 (38.9) | 11 (25.6) |  |  |  |  | |
| **Postoperative adjuvant treatment** |  |  | 1.015  [0.747-1.380] | 0.923 |  |  | |
| Supportive care | 14 (77.8) | 32 (74.4) |  |  |  |  | |
| Chemotherapy | 1 (5.6) | 2 (4.7) |  |  |  |  | |
| Radiotherapy | 0 | 0 |  |  |  |  | |
| Chemoradiotherapy | 3 (16.7) | 7 (16.3) |  |  |  |  | |
| Targeted therapy | 0 | 0 |  |  |  |  | |
| Immunotherapy | 0 | 0 |  |  |  |  | |
| Traditional medicine therapy | 0 | 2 (4.7) |  |  |  |  | |
| OS, overall survival; GBC, gallbladder cancer; OA, open approach; HR, hazards ratio; CI, confidence interval; BMI, body mass index; DM, diabetes mellitus; CA19-9, carbohydrate antigen 19-9; CEA, carcinoembryonic antigen; LNs, lymph nodes. * *P* < 0.1. | | | | | | | |
