# Supplementary material for: Comparison of Outcomes After Primary Laparoscopic Versus Open Approach for T1b/T2 Gallbladder Cancer
Source: Front Oncol. 2021 Oct 28;11:758319. doi: 10.3389/fonc.2021.758319 (PMC8580936; doi:10.3389/fonc.2021.758319)
Supplement: Supplementary file 4 [file Table_2.docx]

| Table S2. Potential risk factors for DFS in GBC patients undergoing OA based on univariable and multivariable analysis. | | | | | | |  |
| --- | --- | --- | --- | --- | --- | --- | --- |
| **Variables** | **Poor OS (n = 18)** | **Good OS (n = 43)** | **Univariable analysis** | | **Multivariable analysis** | | |
|  |  |  | **HR [95%CI]** | ***P* value** | **HR [95%CI]** | ***P* value** | |
| **Demographic data** |  |  |  |  |  |  | |
| Age (years) | 71.5 (46-79) | 60 (42-69) | 1.136  [1.039-1.242] | **0.005*** | 1.095  [1.019-1.177] | **0.013*** | |
| Gender ratio (Male : Female) | 3 : 5 | 5 : 16 | 4.482  [0.574-34.964] | 0.152 |  |  | |
| BMI ≥ 25 kg/m2 | 3 (37.5) | 11 (52.3) | 1.172  [0.946-1.452] | 0.146 |  |  | |
| Smoking | 0 | 4 (19.0) | 3.982  [0.189-84.054] | 0.375 |  |  | |
| DM | 0 | 1 (4.8) | 1.958  [0.031-122.808] | 0.750 |  |  | |
| **Biliary tract disease-related data** |  |  |  |  |  |  | |
| Preoperative jaundice | 0 | 0 | - | - |  |  | |
| Gallbladder stone | 4 (50.0) | 11 (52.4) | 4.380  [1.030-18.629] | **0.046*** | 3.861  [1.426-10.453] | **0.008*** | |
| **Tumor features** |  |  |  |  |  |  | |
| Preoperative CA19-9 (≤ 37 U/ml) | 5 (62.5) | 14 (66.7) | 5.704  [0.605-53.758] | 0.128 |  |  | |
| Preoperative CEA (≤ 5 ng/mL) | 6 (75.0) | 18 (85.7) | 0.516  [0.078-3.399] | 0.492 |  |  | |
| Tumor size (cm) |  |  | 0.329  [0.134-0.807] | **0.015*** | 0.509  [0.278-0.930] | **0.028*** | |
| ≤ 1 | 3 (37.5) | 6 (28.6) |  |  |  |  | |
| 1 - 3 | 4 (50.0) | 7 (33.3) |  |  |  |  | |
| > 3 | 1 (12.5) | 8 (38.1) |  |  |  |  | |
| T stage |  |  | - | - |  |  | |
| T1b | 0 | 0 |  |  |  |  | |
| T2 | 8 (100.0) | 21 (100.0) |  |  |  |  | |
| Positive LNs | 1.375±1.685 | 0.857±1.276 | 1.598  [0.986-2.589] | **0.057*** | 1.430  [1.010-2.023] | **0.044*** | |
| Total harvested LNs | 9.625±3.852 | 10.238±9.219 | 0.988  [0.916-1.066] | 0.759 |  |  | |
| Tumor differentiation |  |  | 0.640  [0.288-1.423] | 0.274 |  |  | |
| Well | 5 (62.5) | 6 (28.6) |  |  |  |  | |
| Moderately | 1 (12.5) | 4 (19.0) |  |  |  |  | |
| Poorly | 2 (25.0) | 11 (52.4) |  |  |  |  | |
| **Postoperative adjuvant treatment** |  |  | 1.189  [0.767-1.845] | 0.439 |  |  | |
| Supportive care | 7 (87.5) | 12 (57.1) |  |  |  |  | |
| Chemotherapy | 0 | 2 (9.5) |  |  |  |  | |
| Radiotherapy | 0 | 0 |  |  |  |  | |
| Chemoradiotherapy | 1 (12.5) | 6 (28.6) |  |  |  |  | |
| Targeted therapy | 0 | 0 |  |  |  |  | |
| Immunotherapy | 0 | 0 |  |  |  |  | |
| Traditional medicine therapy | 0 | 1(4.8) |  |  |  |  | |
| DFS, disease-free survival; GBC, gallbladder cancer; OA, open approach; HR, hazards ratio; CI, confidence interval; BMI, body mass index; DM, diabetes mellitus; CA19-9, carbohydrate antigen 19-9; CEA, carcinoembryonic antigen; LNs, lymph nodes. * *P* < 0.1. | | | | | | | |
